# Adolescent Health in Lebanon: Exploring Alcohol Use, Dietary Patterns, Mental Health, Physical Activity, and Smoking Using the Global School-Based Student Health Survey Approach

**DOI:** 10.3390/nu16213590

**Published:** 2024-10-22

**Authors:** Maha Hoteit, Souheil Hallit, Hanaa Al Rawas, Jana Amasha, Fadia Kobeissi, Rafik Fayyad, Yonna Sacre, Nikolaos Tzenios

**Affiliations:** 1 Food Sciences Unit, National Council for Scientific Research of Lebanon (CNRS-L), Beirut P.O. Box 11-8281, Lebanon; 2 PHENOL Research Program, Faculty of Public Health, Section 1, Lebanese University, Beirut P.O. Box 6573, Lebanon; h.khalifealrawas@st.ul.edu.lb (H.A.R.);; 3 School of Medicine and Medical Sciences, Holy Spirit University of Kaslik, Jounieh P.O. Box 446, Lebanon; souheilhallit@hotmail.com; 4 Department of Psychology, College of Humanities, Effat University, Jeddah 21478, Saudi Arabia; 5 Applied Science Research Center, Applied Science Private University, Amman 11937, Jordan; 6 Department of Social Medical Worker, Faculty of Public Health, Section 1, Lebanese University, Beirut P.O. Box 6573, Lebanon; 7 Department of Nutrition and Food Sciences, Faculty of Arts and Sciences, Holy Spirit University of Kaslik (USEK), Jounieh P.O. Box 446, Lebanon; 8 Faculty of Public Health, Charisma University, London EC1V 7QE, UK

**Keywords:** adolescents, health-related behaviors, Global School-based Student Health Survey, non-communicable diseases

## Abstract

Adolescence is a critical period for establishing lifelong health behaviors; yet in Lebanon, limited data exist on the prevalence of risk factors among this demographic. Objective: This study aims to assess alcohol consumption, dietary habits, physical activity, mental health, and smoking behaviors among Lebanese adolescents aged 13–17 years, with a focus on gender and school-type differences. Methods: A cross-sectional analysis was conducted in Lebanon between March and July 2022 using the Global School-based Student Health Survey (GSHS) questionnaire. A representative sample of students from public and private schools participated in the survey, with key variables analyzed to identify significant patterns and disparities. Results: Our findings reveal that 6.3% of adolescents consumed alcohol, with males reporting a higher prevalence and earlier initiation (*p* = 0.003). Gender differences were evident in dietary habits, where males were more likely to consume sugary drinks (*p* = 0.04) and have consistent breakfast habits (*p* = 0.003). Adolescents from private schools exhibited distinct dietary behaviors, including lower milk consumption (*p* < 0.001) and higher fatty food intake (*p* = 0.008). Males were also more physically active and reported better mental health outcomes compared to females (*p* = 0.004). Smoking behaviors showed that males smoked more frequently, while private school students reported smoking less. No significant difference was observed in bullying experiences between genders or school types. Conclusions: The study highlights critical health behaviors among Lebanese adolescents, with significant variations by gender and school type. These findings underscore the need for targeted interventions to address the identified risk factors and promote healthier behaviors in this population.

## 1. Introduction

Adolescence is a significant change, setting the stage for lifelong health and future success. The choices made during these years—like diet, exercise, mental health, smoking, and social interactions—can have lasting impacts, shaping both physical and emotional well-being. These habits often persist into adulthood, underscoring the importance of this critical phase for personal health and national economic growth [1]. Influenced by peers, families, schools, and communities, adolescents need a united effort from all sectors to foster healthy behaviors and ensure a brighter future [1,2,3]. Since adolescents spend most of their time at school, this crucial environment plays a key role in shaping their lives. Schools are key for socialization and can significantly influence students’ lifestyles. By promoting critical thinking and providing reliable information, schools can help adolescents to identify and reject misinformation [3,4]. Additionally, schools are prime settings for fostering behavior change. Implementing well-organized programs in these institutions can instill healthy habits that last a lifetime [3,4]. 

Lebanon, a low-income country in the Eastern Mediterranean Region, has a population of 5.5 million, including 1.3–1.8 million Syrian and Palestinian refugees [5]. Lebanon has long been a refuge, which has strained its economic and social stability [5]. This ongoing strain has led to widespread mental, physical, and psychological health issues among the population [5]. The coronavirus disease 19 (COVID-19) pandemic, Beirut port explosion, Ukraine–Russia war, and the consequent economic crisis further worsened the country’s health outcomes [6,7,8,9]. 

To help countries to establish effective programs, advocate for resources for school health initiatives, and facilitate cross-national comparisons, the World Health Organization (WHO), in collaboration with The United Nations Children’s Fund (UNICEF), United Nations Educational, Scientific and Cultural Organization (UNESCO), and The Joint United Nations Programme on HIV/AIDS (UNAIDS), and with technical support from the U.S. Centers for Disease Control and Prevention (CDC), launched the Global School-based Student Health Survey (GSHS) in 2003 to evaluate the prevalence of adolescent health-related risk factors, focusing on students aged 13–17. Since the adoption of the Sustainable Development Goals (SDGs) in 2015, there has been a heightened focus on global efforts to reduce Non-Communicable Diseases (NCDs) and their associated risk factors especially among the young population [10]. This survey, conducted worldwide, covers diverse topics, including dietary habits, physical activity, mental health, smoking, alcohol use, and bullying [11]. Lebanon initiated its first GSHS survey in 2005 with the aim of targeting and monitoring the prevalence of critical health risk behaviors and protective factors, identify temporal trends, and establish strategies and interventions to inform decisions, pertaining to school-aged adolescents within the school health program [12]. Since then, no GSHS survey has been conducted until today. Thus, the objective of our study is to (1) assess the prevalence of health risk behaviors among Lebanese adolescents aged 13–17 years using the GSHS questionnaire and approach, and (2) compare the prevalence of these behaviors across gender, age groups, school types, and geographic areas.

## 2. Methods

### 2.1. Study Design and Data Collection

A cross-sectional study including a sample of school-aged adolescents was conducted in Lebanon between March and July 2022. Using the probability cluster sampling technique, a total of 350 participants were included in the study. The study participants were adolescents aged between 13 and 17 years. The clusters from where participants were recruited are the private and public schools from the eight Lebanese governorates (Mount Lebanon, Beirut, South Lebanon, North Lebanon, Akkar, Beqaa, Baalbaeck-Hermel, and Nabatieh). The sample size was calculated using the single population formula a (n = [p (1 − p)] × [(Z∝/2) 2/(e) 2]) where n represents the sample size, Z (∝/2) shows the reliability coefficient of standard error at a 5% level of significance = 1.96, p indicates the likelihood that young people would not be able to practice preventive measures of the diseases (50%), and e refers to the level of standard error tolerated (5%) as stated by Hosmer and Lemeshow [13]. We found that, based on this formula, our sample size is adequate to provide the right amount of power for statistical analysis.

Our research team composed of four dietitians collaborated with numerous schools and municipalities to schedule times and places to meet eligible adolescents with their parents for assessment. After receiving a list of eligible participants, and after receiving the parent’s consent, this study was conducted in two phases:

(i) Phase 1 entailed the adolescents completing an online self-administered questionnaire through email, and (ii) Phase 2 involved performing anthropometric measurements for adolescents at the scheduled study locations by trained dietitians. The online self-administered questionnaire was sent to the participants and filled out in coordination with the trained dietitians one day before the anthropometric data were collected on the scheduled date with the participants.

i. Eligibility of study participants

The participants were chosen based on the following eligibility criteria, which were determined by the study’s purpose: All participants had to be (1) Lebanese nationals, (2) adolescents between the ages of 13 and 17 years, and (3) healthy (no chronic illnesses). Furthermore, we exerted an attempt to contact just one adolescent child from each family.

ii. Study Instrument

A well-designed questionnaire was used to gather data from participants. The first section included 14 questions and asked about date of birth, gender, education level, type of school, residence, primary caregiver, level of education for each parent, employment status, nutrition education in the curriculum, past use of iron dietary supplements, and current use of other dietary supplements (Vitamin D, C, A, B12, folic acid, calcium, magnesium, and zinc). The second segment discussed the GSHS survey; the goal of the GSHS is to gather epidemiological data from students to support school health and youth health programs as well as youth-relevant policies nationally [14] and globally [15]. The GSHS core questionnaire evaluates the following ten modules: “Alcohol use; Dietary behaviors; Drug use; Hygiene; Mental health; Physical activity; Protective factors; Sexual behaviors that contribute to HIV infection, other sexually transmitted infections, and unintended pregnancy; Tobacco use; Violence and unintentional injury” [14]. In this study, six basic modules were selected, including alcohol use (7 questions), dietary behavior (14 questions), physical activity (15 questions), mental health (12 questions), smoking (6 questions), and bullying (7 questions). Note that it is recommended that countries choose at least six of the core modules [15].

### 2.2. Anthropometric Measurements Among Adolescent Participants

Adolescents’ anthropometric measures were taken under standardized conditions by a team of professional dietitians. Every subject had three repetitions of each measurement after which the average value was reported. An electronic scale was used to measure body weight, with a precision of 0.1 kg (Amber Body scale, Numed, Beirut, Lebanon). With subjects barefoot and wearing only the barest minimum of clothing, body weight measurements were taken. Using a portable stadiometer (Portable Height scale, Numed, Beirut, Lebanon), height was measured to the nearest 0.1 cm. Utilizing a measuring tape (Body mass Index (BMI) girth measuring tape, Numed, Beirut, Lebanon), the circumferences of the waist and hips were determined. Adolescents’ waist circumference was measured by wrapping the tape just above their hip bones. The hip width of the adolescent was measured using a tape measure at the widest part of their hip. Using a flexible, non-stretch tape placed midway between the shoulder and elbow, the middle-upper arm circumference (MUAC) was measured to the nearest 0.1 cm.

### 2.3. Ethical Considerations

The study was carried out according to the Helsinki Declaration’s ethical guidelines. The Ethics Committee of the Al-Zahraa University Medical Center, Beirut, Lebanon (Reference Nb 12-2022), provided their ethical approval to conduct this study. Written informed consent was obtained from parents having adolescent children aged less than 18 years old. Participants were fully aware of all the study objectives procedures, from filling out an online questionnaire to doing anthropometric measurements. All measurements were confidential, and the privacy of all participants was guaranteed. Withdrawal from the study was possible at any stage of data collection. In addition, participants’ transportation expenses were reimbursed.

### 2.4. Statistical Analysis

Except for the anthropometric data, the raw data were cleaned and exported for analysis into the Statistical Package of Social Sciences Software (SPSS), IBM Corp., Armonk, NY, USA, version 27.0. To present and compile the study results, descriptive statistical measures such as frequencies, percentages, means, and standard deviations were acquired. An examination of the relationships between the research variables was conducted using a bivariate analysis (χ2 test). In some cases, Fisher’s exact test was utilized in place of a 2 × 2 table where one or more of the cell counts was fewer than 5. All tests were two-sided, and *p*-values of ≤ 0.05 were considered significant.

## 3. Results

### 3.1. Participant’s Sociodemographic Characteristics

Table 1 summarizes the basic socio-demographic characteristics of the study participants. Of the total number of participants (*n* = 350), 51.4% were boys. Most of the adolescents (72.0%) were aged between 13 and 15 years. Most participants were residing in Mount Lebanon (38.3%). A significantly higher percentage of males was aged between 13 and 15 years (*p* = 0.025), lived in Baalbek-Hermel (*p* < 0.001), had both parents as their primary caregivers (*p* = 0.012), and were working (*p* = 0.024) (Table 1). Furthermore, a significantly higher percentage of males were taking folic acid (*p* = 0.026) and vitamin A (*p* = 0.017) (Table 2). 

### 3.2. Anthropometric Characteristics for Adolescents

Table 3 summarizes the anthropometric characteristics of the study participants. A significantly higher mean height (*p* < 0.001), weight (*p* = 0.001), waist to hip ratio (*p* < 0.001), fat free mass (*p* = 0.002), and a lower mean triceps (*p* = 0.011) and biceps (*p* = 0.013) score were seen in males compared to females.

### 3.3. Health Behaviors Among Gender, Age, School Type and Geographic Area

#### 3.3.1. Alcohol Intake

A significantly higher percentage of males drank alcohol (*p* = 0.003), had their first alcoholic drinks before the age of 13 years (*p* = 0.002), and had at least two drinks containing alcohol on three or more days (*p* = 0.010) (Table 4). No significant difference was seen between public and private schools in terms of alcohol drinking (Table 4).

#### 3.3.2. Dietary Behaviors

A significantly higher percentage of males consumed soft drinks on 3 days or less in the last 7 days (*p* = 0.028), had sugary drinks at school (*p* = 0.049), and always had their breakfast in the last 30 days (*p* = 0.003). In addition, participants from private schools had never had milk and milk products in the last 7 days (*p* < 0.001), consumed fatty food more than three times in the last 7 days (*p* = 0.008), sweets three times or less in the last 7 days (*p* = 0.047), never ate fast food (*p* = 0.005) or drank soft drinks (*p* = 0.039) in the last 7 days, and did not receive sugary drinks at school (*p* < 0.001) (Table 5). In our findings, we observed notable differences in dietary behaviors between private and public school students, particularly regarding the consumption of fatty foods and fast foods. Private school students reported a higher intake of processed meats, this included items such as sausages, bacon, and various deli meats, which are often high in saturated fats. In addition to full-fat dairy products, many students consumed whole milk, full-fat cheeses, and cream-based products, contributing to their overall fat intake. Moreover, snack foods that included potato chips, pastries, and cookies, which tend to be high in unhealthy fats and sugars, were also recorded. As for the fried foods such as fried chicken, french fries, and other deep-fried snacks, students were asked about these. All these items were listed under the category of fatty foods according to the questionnaire. Interestingly, our study revealed that private-school students had a lower consumption of fast-food items compared to their public-school peers. Fast food categories where we noted a lower intake included burgers and fries, pizza, and fast-casual dining. All these categories were included in the question regarding fast foods in the questionnaire. Regarding the provision of in-school meal programs, most (>70%) private and public schools do not provide meal programs especially breakfast (*p* < 0.001).

#### 3.3.3. Physical Activity

A significantly higher percentage of males were active for 60 min on 3 or more days (*p* < 0.001), engaged in team sports (*p* < 0.001), and had 8 or more sleeping hours per day (*p* = 0.003). In addition, a significantly a higher percentage of adolescents from private schools did not engage in a sports team (*p* < 0.001), never did stretching exercises (*p* < 0.001), did not have ground activities after school (*p* = 0.004), and did not attend classes about the benefit of physical activity (*p* < 0.001) (Table 6).

#### 3.3.4. Mental Health Indicators

A significantly higher percentage of males did not feel lonely (*p* = 0.004), were never worried about something that they could not sleep at night (*p* < 0.001) and never felt nervous or anxious or not able to stop or control worrying (*p* < 0.001) in the last 12 months. Moreover, a significantly higher percentage of adolescents from private schools felt lonely most of the time or always (*p* = 0.035), had less than two close friends (*p* = 0.002), and did not take classes about anger management (*p* < 0.001) (Table 7).

#### 3.3.5. Tobacco Use

A significantly higher percentage of males smoked for 3 or more days in the last 30 days (*p* = 0.044), never used tobacco products other than cigarettes (*p* = 0.001). Furthermore, a significantly higher percentage of adolescents from private schools smoked for less than 3 days in the last 30 days (Table 8).

#### 3.3.6. Bullying Experiences

No significant difference was found between males and females and between participants from public and private schools in terms of bullying (Table 9).

## 4. Discussion

### 4.1. Alcohol Intake

Our findings showed that 6.3% of adolescents consumed alcohol. This is less than the report published previously by Pengpid et al. showing that the prevalence of alcohol use in Lebanon was 27.8% among boys and 12.2% among girls in 2005 and did not change significantly over the years between 2011 and 2015 [16]. This could be explained by the decline in purchasing power among Lebanese households due to the financial and economic crisis [6,17]. In our study, a significantly higher percentage of male students reported alcohol consumption compared to females (*p* = 0.003), with males also more likely to have had their first alcoholic drink before the age of 13 (*p* = 0.002) and to consume at least two alcoholic drinks on three or more days (*p* = 0.010). These findings align with global trends observed in the Global School-based Student Health Survey (GSHS), where male students generally report higher rates of alcohol consumption and earlier initiation than their female counterparts [18]. Similarly, in the Arab region, although alcohol consumption among adolescents tends to be lower due to cultural and religious factors, when it does occur, males are typically more likely to drink and to start drinking at a younger age [19]. Interestingly, our study found no significant difference in alcohol consumption between students from public and private schools, suggesting that the type of school does not influence drinking behavior in Lebanon. On the other hand, research on the differences in alcohol consumption between students in private and public schools reveals mixed findings, which vary by region. In the United States, some studies suggest that private school students are more likely to consume alcohol due to higher socioeconomic status and social environments that may normalize alcohol use [20,21]. In Europe, while some studies indicate that private school students engage in higher levels of alcohol consumption, others find no significant differences, suggesting that peer influence and parental monitoring might play a more crucial role [22]. In the Middle East and North Africa (MENA) region, including Lebanon, the cultural context often places strong social restrictions on alcohol use, which might minimize differences between public and private school students [19]. Supporting this, a study by Karam et al. (2010) in Lebanon found no significant difference in alcohol consumption between these two groups, consistent with our findings [23]. These varied results highlight the complexity of factors influencing adolescent drinking behavior, with school type being just one of many contributing elements.

### 4.2. Dietary Intake

Our study reveals significant gender and school-type differences in dietary habits among adolescents. Males were more likely to consume soft drinks on three days or less in the last seven days (*p* = 0.028), to have sugary drinks at school (*p* = 0.049), and to have had breakfast consistently over the last 30 days (*p* = 0.003). These patterns suggest that male students may have a higher exposure to sugary beverages at school and possibly better breakfast habits. Globally, dietary habits among adolescents vary significantly, often influenced by factors such as gender, socioeconomic status, and school environment. Studies have shown that male students typically consume more sugary beverages, which aligns with our findings [24]. Additionally, the trend of male students being more consistent with breakfast consumption is observed globally, as breakfast is often associated with a better academic performance and overall health, which might be more emphasized for boys in certain cultures [25]. In contrast, students from private schools exhibited distinct dietary behaviors, with a significantly higher likelihood of never consuming milk or milk products in the last seven days (*p* < 0.001), consuming fatty foods more than three times in the last seven days (*p* = 0.008), and eating sweets three times or less in the last seven days (*p* = 0.047). Interestingly, these students were also more likely to report never eating fast food (*p* = 0.005), not drinking soft drinks (*p* = 0.039), not receiving sugary drinks at school (*p* < 0.001), and never having breakfast provided by their school (*p* < 0.001). These findings contrast with some global trends where private school students, often from more affluent backgrounds, typically have higher access to and consumption of dairy products and may engage more in fast food and soft drink consumption [26]. The lower milk intake and higher fatty food consumption observed in our study could reflect cultural preferences or shifts toward non-traditional diets in wealthier Lebanese families [17,27]. Regionally, in the Arab world, the high consumption of fatty foods aligns with traditional dietary patterns rich in fats, although the lower consumption of fast food and sugary drinks might indicate greater health awareness among private school students [28]. These patterns suggest the influence of cultural, socioeconomic, and educational factors in shaping the dietary habits of adolescents in private schools, highlighting the need for targeted nutritional interventions that consider these complex influences. 

In the Arab region, dietary habits among adolescents also reflect cultural and socioeconomic influences. Research in countries like Jordan and Saudi Arabia has shown that males are more likely to consume soft drinks and sugary beverages, particularly in school settings [29,30,31]. The dietary behaviors of private school students in our study, such as a higher consumption of fatty foods and lower consumption of fast food, may reflect different nutritional environments and health education practices compared to public schools. These findings underscore the need for targeted nutritional interventions that consider both gender and the type of school, especially in private school settings where certain unhealthy dietary patterns may be more prevalent.

### 4.3. Physical Activity

Our study indicates significant differences in physical activity and related behaviors based on gender and school type among adolescents. A significantly higher percentage of males were active for at least 60 min on three or more days (*p* < 0.001), participated in team sports (*p* < 0.001), and achieved eight or more hours of sleep per day (*p* = 0.003). These findings are consistent with global research, which generally shows that male adolescents are more likely to engage in physical activities and team sports due to social norms and the greater encouragement of physical exercise for boys [32]. The importance of sleep in relation to physical activity is also well-documented, with studies indicating that active adolescents, particularly males, are more likely to maintain healthy sleep patterns [33]. In contrast, adolescents from private schools were less likely to participate in team sports (*p* < 0.001), never did stretching exercises (*p* < 0.001), did not participate in ground activities after school (*p* = 0.004), and did not attend classes about the benefits of physical activity (*p* < 0.001). This trend aligns with findings from other studies in the Arab region, where students from higher socioeconomic backgrounds, often attending private schools, may face more academic pressures and have less time or encouragement to engage in physical activities [34,35]. The lack of physical education and activity-focused classes in private schools could reflect different educational priorities that emphasize academic achievement over physical health. 

### 4.4. Mental Health Indicators

Our study highlights notable differences in mental health indicators based on gender and school type among adolescents. A significantly higher percentage of males reported not feeling lonely (*p* = 0.004), never experiencing sleep disturbances due to worry (*p* < 0.001), and not feeling nervous, anxious, or unable to control worrying in the past year (*p* < 0.001). These findings are consistent with global trends where males generally report lower levels of emotional distress compared to females. Research indicates that societal norms and coping mechanisms often result in males exhibiting fewer symptoms of anxiety and loneliness [36]. This pattern is supported by studies showing that males are less likely to report feelings of sadness or anxiety, potentially due to differences in emotional expression and support systems [37]. Conversely, adolescents from private schools were more likely to report feeling lonely most of the time or always (*p* = 0.035), having fewer than two close friends (*p* = 0.002), and not participating in anger management classes (*p* < 0.001). This trend mirrors findings in the Arab region, where students in private schools may experience higher levels of social isolation and loneliness [38]. Research in the Middle East suggests that students in more affluent, competitive environments often face increased emotional stress and fewer opportunities for social engagement due to rigorous academic demands and social pressures [38]. The lack of anger management classes in these settings may further exacerbate feelings of isolation and stress. 

### 4.5. Tobacco Use

Our study reveals significant gender and school-type differences in smoking behaviors among adolescents. A notably higher percentage of males reported smoking for three or more days in the last 30 days (*p* = 0.044) and never using tobacco products other than cigarettes (*p* = 0.001). This finding aligns with European research indicating that males are generally more likely to engage in cigarette smoking compared to females, often due to social norms and greater peer influences related to smoking [39]. The higher prevalence of cigarette smoking among males may reflect underlying behavioral patterns and risk factors unique to this demographic. Conversely, adolescents from private schools were significantly more likely to report smoking for fewer than 3 days in the last 30 days. This contrasts with some global patterns where students in private schools, often from more affluent backgrounds, might have higher access to smoking-related products and potentially face greater social influences on smoking [40]. However, in the context of Lebanon, this trend could suggest that private school students, who might be exposed to stricter health education and anti-smoking campaigns, exhibit a lower smoking frequency compared to their peers in other school types. This could be reflective of the preventive measures and health promotion efforts implemented in these schools, highlighting the importance of targeted anti-smoking education programs that can influence smoking behaviors across different educational environments.

### 4.6. Bullying Experiences

Our study found no significant difference in bullying experiences between males and females, nor between students from public and private schools. This lack of gender difference in bullying aligns with some global research, which suggests that bullying behavior can be equally prevalent among both genders, though the forms of bullying may differ. Males are often associated with more physical forms of bullying, while females may engage in relational or verbal bullying, leading to similar overall prevalence rates [41]. The absence of a significant difference in bullying between public and private school students is also noteworthy. In some contexts, private schools may have more resources to address bullying through anti-bullying programs and support services, potentially leading to lower reported rates. However, our findings suggest that bullying remains a pervasive issue across different school environments in Lebanon, regardless of socioeconomic status or school type. Regionally, research in the Arab world has produced mixed findings on bullying, with some studies indicating higher rates in public schools due to larger class sizes and less supervision, while others report no significant differences between school types, similar to our study [42]. This suggests that bullying is a complex issue influenced by various factors, including cultural norms, school policies, and the effectiveness of anti-bullying interventions. The lack of difference in bullying prevalence between public and private schools in Lebanon may reflect a broader need for comprehensive anti-bullying strategies that are effective across all educational settings.

### 4.7. Holistic Approaches to Adolescent Health: Understanding Interconnected Behaviors in the Lebanese Context

Our findings underscore the intricate interconnectedness of various health behaviors and risk factors among Lebanese adolescents. Specifically, alcohol consumption, dietary habits, physical activity, mental health, tobacco use, and bullying experiences do not exist in isolation; rather, they are intertwined, each influencing and reinforcing the others [43]. For instance, unhealthy dietary habits may lead to lower physical activity levels, which in turn can negatively affect mental health [44]. Similarly, high levels of alcohol consumption may correlate with increased tobacco use and risky behaviors, while experiences of bullying can exacerbate mental health challenges, further complicating an adolescent’s overall well-being [45]. The cultural and socioeconomic landscape of Lebanon plays a pivotal role in shaping these behaviors and risk factors. Cultural norms surrounding alcohol and tobacco use, as well as dietary preferences, are deeply embedded in Lebanese society and can differ significantly based on gender and school type [46]. For example, boys may be more likely to engage in riskier behaviors such as drinking and smoking, influenced by societal expectations of masculinity, while private school students might experience unique pressures related to academic achievement that impact their physical activity and mental health. The economic crisis further exacerbates these issues, affecting access to healthy food, recreational activities, and mental health resources, thereby amplifying disparities across different demographic groups. To effectively improve the overall health and well-being of adolescents, it is essential to implement comprehensive strategies that address these interconnected factors holistically. Interventions should not treat each behavior in isolation; instead, they must recognize the complex relationships between them. For instance, programs that promote healthy eating should also incorporate physical activity initiatives and mental health support, creating a more integrated approach to adolescent health. By fostering a deeper understanding of these complex interrelations, public health initiatives and educational programs can be more effectively tailored to meet the diverse needs of youth in Lebanon. This integrated perspective can ultimately lead to the promotion of healthier lifestyles and the reduction in risk factors associated with poor health outcomes. Recognizing the collective impact of these behaviors will be crucial in informing better public health policies and interventions, paving the way for a healthier future for Lebanese adolescents.

### 4.8. Limitations and Strengths

While our study offers valuable insights, it is important to acknowledge the inherent limitations that define its scope. One key limitation is the exclusion of several important modules from the ten core modules of the GSHS, recommended by the WHO, such as those on violence, drug use, and sexual behavior. The sexual behavior module was excluded due to ethical concerns. Additionally, the reliance on self-reported data introduces potential biases, such as underreporting or overreporting by participants. Moreover, the data collection in this survey was not based on the Ministry of Education’s connections with schools. Despite these limitations, our research stands out as the first study to specifically examine health-related risk behaviors among adolescents across different age groups and geographic areas in Lebanon. The survey’s standardized questions and methodology enable reliable comparisons, both within Lebanon and with other countries conducting studies using the GSHS approach. 

## 5. Conclusions

Our study highlights critical patterns in adolescent health behaviors in Lebanon, revealing that 6.3% of adolescents consumed alcohol, with males showing a higher prevalence and earlier initiation. Significant gender and school-type differences were found in dietary habits, physical activity, and mental health indicators, with males generally engaging more in physical activities and reporting better mental health outcomes. Additionally, smoking behaviors varied by gender and school type, with males smoking more frequently. These findings underscore the need for targeted public health interventions to address these risk behaviors among Lebanese adolescents.

## Figures and Tables

**Table 1:** Sociodemographic characteristic of the study population, overall and by gender.

		Overall (350)		Female (170, 48.6%)		Male (180, 51.4%)		*p*-Value
		n	%	n	%	n	%	
Age categories								0.025
	13–15	252	72	113	66.5	139	77.2	
	16–17	98	28	57	33.5	41	22.8	
Residence								0.223
	Beirut and Mount Lebanon	145	41.4	65	38.2	80	44.4	
	North Lebanon/Akkar	73	20.9	41	24.1	32	17.8	
	South Lebanon/Nabatieh	85	24.3	45	26.5	40	22.2	
	Beqaa and Baalbek-Hermel	47	13.4	19	11.2	28	15.6	
Education level								0.3
	Elementary school level (Grades 1–6)	115	32.9	56	32.9	59	32.8	
	Intermediate school level (Grades 7–9)	123	35.1	54	31.8	69	38.3	
	Secondary school level Grades 10–12)/vocational	112	32.0	60	35.3	52	28.9	
School type								0.3
	Public school	168	48.0	77	45.3	91	50.6	
	Private school	182	52.0	93	54.7	89	49.4	
Primary caregiver								0.01
	Mom	5	1.4	5	2.9	0	0	
	Dad	4	1.1	2	1.2	2	1.1	
	Both	334	95.4	157	92.4	177	98.3	
	Others	7	2.0	6	3.5	1	0.6	
Education level of mother								0.2
	Illiterate	27	7.7	9	5.3	18	10.0	
	Elementary school level (Grades 1–6)	51	14.6	23	13.5	28	15.6	
	Intermediate school level (Grades 7–9)	94	26.9	53	31.2	41	22.8	
	Secondary school level Grades 10–12)/vocational	75	21.4	37	21.8	38	21.1	
	University level	103	29.4	48	28.2	55	30.6	
Education level of father								0.2
	Illiterate	35	10.0	16	9.4	19	10.6	
	Elementary school level (Grades 1–6)	71	20.3	34	20.0	37	20.6	
	Intermediate school level (Grades 7–9)	92	26.3	51	30.0	41	22.8	
	Secondary school level Grades 10–12)/vocational	80	22.9	31	18.2	49	27.2	
	University level	72	20.6	38	22.4	34	18.9	
Working status								0.02
	No	335	95.7	167	98.2	168	93.3	
	Yes	15	4.3	3	1.8	12	6.7	
Nutrition education in school curriculum								0.7
	No	317	90.6	153	90.0	164	91.1	
	Yes	33	9.4	17	10.0	16	8.9	

**Table 2:** Prevalence of dietary intake supplements intake, overall and by gender.

			Gender				
	Overall (350)		Female (170, 48.6%)		Male (180, 51.4%)		
	N	%	N	%	N	%	*p*-Value
Previous iron intake							0.353
No	273	78.0	129	75.9	144	80.0	
Yes	77	22.0	41	24.1	36	20.0	
Zinc intake							0.122
No	326	93.1	162	95.3	164	91.1	
Yes	24	6.9	8	4.7	16	8.9	
Magnesium intake							0.284
No	324	92.6	160	94.1	164	91.1	
Yes	26	7.4	10	5.9	16	8.9	
Calcium intake							0.059
No	324	92.6	162	95.3	162	90.0	
Yes	26	7.4	8	4.7	18	10.0	
Folic acid							0.026
No	327	93.4	164	96.5	163	90.6	
Yes	23	6.6	6	3.5	17	9.4	
Vitamin A							0.017
No	326	93.1	164	96.5	162	90.0	
Yes	24	6.9	6	3.5	18	10.0	
Vitamin C							0.084
No	313	89.4	157	92.4	156	86.7	
Yes	37	10.6	13	7.6	24	13.3	
Vitamin B12							0.231
No	321	91.7	159	93.5	162	90.0	
Yes	29	8.3	11	6.5	18	10.0	
Vitamin D							0.933
No	295	84.3	143	84.1	152	84.4	
Yes	55	15.7	27	15.9	28	15.6	

**Table 3:** Anthropometric characteristics of the study population, overall and by gender.

	Overall (n = 350)	Girls (n = 170)	Boys (n = 180)	
	Mean ± SD	Mean ± SD	Mean ± SD	*p*-Value
Body weight (kg)	50.83 ± 17.80	48.95 ± 15.25	52.60 ± 19.78	0.054
Height (cm)	154.18 ± 13.03	151.76 ± 10.42	156.46 ± 14.76	<0.001
Waist (cm)	69.82 ± 12.96	67.55 ± 11.80	71.98 ± 13.66	0.001
Hip (cm)	86.57 ± 13.31	87.15 ± 12.80	86.04 ± 13.78	0.437
Waist to hip ratio	0.81 ± 0.07	0.78 ± 0.07	0.83 ± 0.06	<0.001
MUAC (cm)	23.57 ± 4.49	23.39 ± 4.49	23.74 ± 4.69	0.480
Sum skinfold (cm)	47.68 ± 26.09	49.69 ± 24.03	45.80 ± 27.81	0.163
Percentage of body fat	24.04 ± 8.09	24.90 ± 7.52	23.24 ± 8.54	0.055
Total fat mass (kg)	13.20 ± 9.02	13.08 ± 8.15	13.31 ± 9.79	0.815
Fat free mass (Kg)	37.61 ± 10.60	35.83 ± 8.43	39.29 ± 12.07	0.002
Triceps in cm	12.61 ± 6.15	13.46 ± 5.50	11.81 ± 6.63	0.011
Biceps in cm	8.16 ± 4.68	8.79 ± 4.60	7.56 ± 4.69	0.013
Subscapular in mm	13.60 ± 8.64	13.99 ± 8.33	13.24 ± 8.92	0.415
Supra-iliac in mm	13.31 ± 8.90	13.43 ± 8.12	13.21 ± 9.61	0.817

**Table 4:** Alcohol intake, overall, by gender and by school type.

		Overall (*n* = 350)		Gender					School Type				
				Female(n = 170)		Male(n = 180)			Public (n = 167)		Private (n = 185)		
		N	%	N	%	N	%	*p*-Value	N	%	N	%	*p*- Value
Drink alcohol	0.003								0.050				
	No	328	93.7	166	97.6	162	90.0		153	91.1	175	96.2	
	Yes	22	6.3	4	2.4	18	10.0		15	8.9	7	3.8	
Age for first alcoholic drink		0.002							0.144				
	Never drink alcohol	328	93.7	166	97.6	162	90.0		153	91.1	175	96.2	
	13 years old or before	17	4.9	2	1.2	15	8.3		12	7.1	5	2.7	
	14 years old and older	5	1.4	2	1.2	3	1.7		3	1.8	2	1.1	
During the past 30 days, on how many days did you have at least 2 drinks containing alcohol?		0.010							0.340				
	Never	336	96.0	167	98.2	169	93.9		159	94.6	177	97.3	
	Less than 3 days	6	1.7	3	1.8	3	1.7		3	1.8	3	1.6	
	3 days or more	8	2.3	0	0	8	4.4		6	3.6	2	1.1	
During the past 30 days, on the days you drank alcohol, how many drinks did you usually drink per day?	0.08								0.3				
	I did not drink alcohol	336	96.0	167	98.2	169	93.9		159	94.6	177	97.3	
	Less than 3 drinks	12	3.4	3	1.8	9	5.0		7	4.2	5	2.7	
	3 drinks or more	2	0.6	0	0	2	1.1		2	1.2	0	0	
During the past 30 days, how did you usually obtain the alcohol you drank?								0.18					0.3
	I did not drink alcohol	337	96.3	167	98.2	170	94.4		160	95.2	177	97.3	
	Store, shop, or from a street vendor	5	1.4	2	1.2	3	1.7		4	2.4	1	0.5	
	I got it from my family	7	2.0	1	0.6	6	3.3		3	1.8	4	2.2	
	Other way	1	0.3	0	0	1	0		1	0.6	0	0	
How many times have you gotten into trouble with your family or friends, or gotten into fights, after drinking alcohol?								1					1
	Never	348	99.4	169	99.4	179	99.4		167	99.4	181	99.5	
	1 or 2 times	2	0.6	1	0.6	1	0.6		1	0.6	1	0.5	
How many times have you drunk so much alcohol that you were really drunk?								0.2					0.8
	Never	343	98.0	169	99.4	174	96.7		164	97.6	179	98.4	
	Less than 3 times	2	0.6	0	0	2	1.1		1	0.6	1	0.5	
	3 times or more	5	1.4	1	0.6	4	2.2		3	1.8	2	1.1	

**Table 5:** Dietary behaviors, overall, by gender and school type.

				Gender					School Type				
		Overall (n = 350)		Female (n = 170)		Male (n = 180)			Public (n = 167)		Private (n = 185)		
		N	%	N	%	N	%	*p*-Value	N	%	N	%	*p*-Value
During the past 30 days, how often did you go hungry because there was not enough food in your home?								0.241					0.549
	Never	229	65.4	104	61.2	125	69.4		107	63.7	122	67.0	
	Rarely/Sometimes	108	30.9	58	34.1	50	27.8		56	33.3	52	28.6	
	Most of the time/Always	13	3.7	8	4.7	5	2.8		5	3.0	8	4.4	
Milk and milk products (last 7 days)								0.08					<0.001
	Never	70	20.0	40	23.5	30	16.7		23	13.7	47	25.8	
	3 or fewer times during the past 7 days	143	40.9	60	35.3	83	46.1		57	33.9	86	47.3	
	More than 3 times during the past 7 days	137	39.1	70	41.2	67	37.2		88	52.4	49	26.9	
Fatty food (last 7 days)								0.053					0.008
	Never	62	17.7	28	16.5	34	18.9		28	16.7	34	18.7	
	3 or fewer times during the past 7 days	150	42.9	64	37.6	86	47.8		86	51.2	64	35.2	
	More than 3 times during the past 7 days	138	39.4	78	45.9	60	33.3		54	32.1	84	46.2	
Sweets (last 7 days)								0.09					0.04
	Never	32	9.1	12	7.1	20	11.1		14	8.3	18	9.9	
	3 or fewer times during the past 7 days	144	41.1	64	37.6	80	44.4		59	35.1	85	46.7	
	More than 3 times during the past 7 days	174	49.7	94	55.3	80	44.4		95	56.5	79	43.4	
Fiber-rich food (last 7 days)								0.98					0.91
	Never	45	12.9	22	12.9	23	12.8		22	13.1	23	12.6	
	3 or fewer times during the past 7 days	153	43.7	75	44.1	78	43.3		75	44.6	78	42.9	
	More than 3 times during the past 7 days	152	43.4	73	42.9	79	43.9		71	42.3	81	44.5	
Fast food (last 7 days)								0.82					0.005
	Never	278	79.4	133	78.2	145	80.6		122	72.6	156	85.7	
	Less than 3 days	46	13.1	23	13.5	23	12.8		27	16.1	19	10.4	
	3 or more days	26	7.4	14	8.2	12	6.7		19	11.3	7	3.8	
Soft drinks (last 7 days)								0.02					0.03
	Never	167	47.7	79	46.5	88	48.9		71	42.3	96	52.7	
	3 or fewer times	112	32.0	47	27.6	65	36.1		54	32.1	58	31.9	
	More than 3 times during the past 7 days	71	20.3	44	25.9	27	15.0		43	25.6	28	15.4	
Receive sugary drinks at school								0.04					<0.001
	No	229	65.4	120	70.6	109	60.6		94	56.0	135	74.2	
	Yes	121	34.6	50	29.4	71	39.4		74	44.0	47	25.8	
Breakfast intake (last 30 days)								0.003					0.44
	Never	26	7.4	16	9.4	10	5.6		8	4.8	18	9.9	
	Rarely	33	9.4	22	12.9	11	6.1		16	9.5	17	9.3	
	Sometimes	60	17.1	37	21.8	23	12.8		28	16.7	32	17.6	
	Most of the time	62	17.7	28	16.5	34	18.9		30	17.9	32	17.6	
	Always	169	48.3	67	39.4	102	56.7		86	51.2	83	45.6	
Reason to skip breakfast								0.003					0.35
	I always eat breakfast	216	61.7	89	52.4	127	70.6		106	63.1	110	60.4	
	I cannot eat early in the morning	64	18.3	42	24.7	22	12.2		33	19.6	31	17.0	
	I do not have time for breakfast	21	6.0	9	5.3	12	6.7		8	4.8	13	7.1	
	There is not always food in my home	18	5.1	10	5.9	8	4.4		5	3.0	13	7.1	
	Always	31	8.9	20	11.8	11	6.1		16	9.5	15	8.2	
Breakfast provided by school								0.06					<0.001
	Never	286	81.7	130	76.5	156	86.7		124	73.8	162	89.0	
	Rarely	21	6.0	16	9.4	5	2.8		14	8.3	7	3.8	
	Sometimes	10	2.9	5	2.9	5	2.8		4	2.4	6	3.3	
	Most of the time	12	3.4	7	4.1	5	2.8		10	6.0	2	1.1	
	Always	21	6.0	12	7.1	9	5.0		16	9.5	5	2.7	

**Table 6:** Physical activity, overall, by gender and school type.

				Gender					School Type				
		Overall (n = 350)		Female (n = 170)		Male (n = 180)			Public School (n = 167)		Private School (n = 185)		
		N	%	N	%	N	%	*p*-Value	N	%	N	%	*p*-Value
Days being active for 60 min								<0.001					0.621
	Never	87	24.9	48	28.2	39	21.7		41	24.4	46	25.3	
	Less than 3 days	82	23.4	54	31.8	28	15.6		36	21.4	46	25.3	
	3 or more days	181	51.7	68	40.0	113	62.8		91	54.2	90	49.5	
Walking or bicycling to or from school (last 7)								0.251					0.113
	Never	211	60.3	97	57.1	114	63.3		101	60.1	110	60.4	
	Less than 3 days	50	14.3	23	13.5	27	15.0		30	17.9	20	11.0	
	3 or more days	89	25.4	50	29.4	39	21.7		37	22.0	52	28.6	
Engaged in sport teams								<0.001					<0.001
	No	235	67.1	133	78.2	102	56.7		93	55.4	142	78.0	
	Yes	115	32.9	37	21.8	78	43.3		75	44.6	40	22.0	
Stretching exercises (last 7 days)								0.299					<0.001
	Never	257	73.4	125	73.5	132	73.3		98	58.3	159	87.4	
	Less than 3 days	46	13.1	26	15.3	20	11.1		38	22.6	8	4.4	
	3 or more days	47	13.4	19	11.2	28	15.6		32	19.0	15	8.2	
Ground activities after school								<0.001					0.004
	No	207	59.1	131	77.1	76	42.2		86	51.2	121	66.5	
	Yes	143	40.9	39	22.9	104	57.8		82	48.8	61	33.5	
Sitting time per day								0.598					0.329
	Less than 4 h per day	228	65.1	110	64.7	118	65.6		114	67.9	114	62.6	
	4–8 h per day	98	28.0	46	27.1	52	28.9		41	24.4	57	31.3	
	More than 8 h per day	24	6.9	14	8.2	10	5.6		13	7.7	11	6.0	
Sleeping hours								0.003					0.742
	Less than 8 h	149	42.6	86	50.6	63	35.0		70	41.7	79	43.4	
	8 h or more per day	201	57.4	84	49.4	117	65.0		98	58.3	103	56.6	
How many times did you see, read or listen to encouragement advertisements about PA								0.079					0.400
	Never	186	53.1	89	52.4	97	53.9		83	49.4	103	56.6	
	Rarely/Sometimes	142	40.6	75	44.1	67	37.2		74	44.0	68	37.4	
	Almost daily/Daily	22	6.3	6	3.5	16	8.9		11	6.5	11	6.0	
Classes about the benefits of physical activity (during this school year)							0.365						<0.001
	No	262	74.9	132	77.6	130	72.2		109	64.9	153	84.1	
	Yes	56	16.0	26	15.3	30	16.7		42	25.0	14	7.7	
	I do not know	32	9.1	12	7.1	20	11.1		17	10.1	15	8.2	

**Table 7:** Mental health indicators, overall, by gender and school type.

				Gender					School Type				
		Overall (n = 350)		Female (n = 170)		Male (n = 180)			Public (n = 167)		Private (n = 185)		
		N	%	N	%	N	%	*p*-Value	N	%	N	%	*p*-Value
Feeling lonely (last 12 months)								0.004					0.035
	Never	166	47.4	65	38.2	101	56.1		73	43.5	93	51.1	
	Rarely	69	19.7	35	20.6	34	18.9		44	26.2	25	13.7	
	Sometimes	76	21.7	47	27.6	29	16.1		34	20.2	42	23.1	
	Most of the time or always	39	11.1	23	13.5	16	8.9		17	10.1	22	12.1	
Worried about something so much that you could not sleep at night (last 12 months)								<0.001					0.540
	Never	159	45.4	62	36.5	97	53.9		72	42.9	87	47.8	
	Rarely	94	26.9	46	27.1	48	26.7		51	30.4	43	23.6	
	Sometimes	75	21.4	42	24.7	33	18.3		34	20.2	41	22.5	
	Most of the time or always	22	6.3	20	11.8	2	1.1		11	6.5	11	6.0	
Considering suicide seriously (last 12 months)								0.834					0.433
	No	339	96.9	165	97.1	174	96.7		164	97.6	175	96.2	
	Yes	11	3.1	5	2.9	6	3.3		4	2.4	7	3.8	
Number of close friends								0.025					0.002
	Less than 2 friends	73	20.9	44	25.9	29	16.1		23	13.7	50	27.5	
	2 or more friends	277	79.1	126	74.1	151	83.9		145	86.3	132	72.5	
Worried about something so much that you could not eat, did not feel hungry, or ate too much (last 12 months)								<0.001					0.389
	Never	197	56.3	81	47.6	116	64.4		94	56.0	103	56.6	
	Rarely	64	18.3	31	18.2	33	18.3		34	20.2	30	16.5	
	Sometimes	72	20.6	43	25.3	29	16.1		35	20.8	37	20.3	
	Most of the time/always	17	4.9	15	8.8	2	1.1		5	3.0	12	6.6	
Feel down, depressed, or hopeless or have little interest in or do not get much pleasure from doing things (last 12 months)								0.120					0.554
	Never	159	45.4	66	38.8	93	51.7		81	48.2	78	42.9	
	Rarely	76	21.7	41	24.1	35	19.4		38	22.6	38	20.9	
	Sometimes	77	22.0	42	24.7	35	19.4		32	19.0	45	24.7	
	Most of the time/always	38	10.9	21	12.4	17	9.4		17	10.1	21	11.5	
Feel nervous or anxious or not able to stop or control worrying (last 12 months)								<0.001					0.094
	Never	157	44.9	58	34.1	99	55.0		65	38.7	92	50.5	
	Rarely	97	27.7	46	27.1	51	28.3		53	31.5	44	24.2	
	Sometimes	59	16.9	33	19.4	26	14.4		28	16.7	31	17.0	
	Most of the time/always	37	10.6	33	19.4	4	2.2		22	13.1	15	8.2	
Have a hard time staying focused on your homework (last 12 months)								0.325					0.550
	Never	130	37.1	55	32.4	75	41.7		59	35.1	71	39.0	
	Rarely	124	35.4	65	38.2	59	32.8		61	36.3	63	34.6	
	Most of the time/always	75	21.4	40	23.5	35	19.4		40	23.8	35	19.2	
What method did you use for the most recent suicide attempt?								0.070					0.139
	I did not attempt suicide	342	97.7	165	97.1	177	98.3		167	99.4	175	96.2	
	I cut myself	4	1.1	4	2.4	0	0		1	0.6	3	1.6	
	I used some other method	4	1.1	1	0.6	3	1.7		0	0	4	2.2	
Classes on how to manage anger (this school year)								0.138					<0.001
	No	290	82.9	146	85.9	144	80.0		126	75.0	164	90.1	
	Yes	34	9.7	11	6.5	23	12.8		23	13.7	11	6.0	
	I do not know	26	7.4	13	7.6	13	7.2		19	11.3	7	3.8	

**Table 8:** Tobacco use, overall, by gender and school type.

				Gender					School Type				
		Overall (n = 350)		Female (n = 172)		Male (n = 180)			Public (n = 167)		Private (n = 185)		
		N	%	N	%	N	%	*p*-Value	N	%	N	%	*p*-Value
Smoking	0.275												0.638
	No	295	84.3	147	49.8	148	50.2		140	47.5	155	52.5	
	Yes	55	15.7	23	41.8	32	58.2		28	50.9	27	49.1	
Age for first smoking attempt								0.278					0.374
	I have never smoked cigarettes	296	84.6	147	49.7	149	50.3		141	47.6	155	52.4	
	Less than 13 years old	29	8.3	10	34.5	19	65.5		12	41.4	17	58.6	
	Older than 13 years	25	7.1	13	52.0	12	48.0		15	60.0	10	40.0	
Days did you smoke cigarettes (last 30 days)								0.044					0.013
	Never	333	95.1	166	49.8	167	50.2		157	47.1	176	52.9	
	Less than 3	7	2.0	3	42.9	4	57.1		2	28.6	5	71.4	
	3 days or more	10	2.9	1	10.0	9	90.0		9	90.0	1	10.0	
Days of using tobacco products other than cigarettes (last 30 days)								0.001					0.670
	Never	323	92.3	148	45.8	175	54.2		156	48.3	167	51.7	
	Less than 3 days	9	2.6	7	77.8	2	22.2		5	55.6	4	44.4	
	3 days or more	18	5.1	15	83.3	3	16.7		7	38.9	11	61.1	
Days of using smokeless tobacco products (last 30 days)								0.802					0.797
	Never	346	98.9	168	48.6	178	51.4		166	48.0	180	52.0	
	Less than 6 days	1	0.3	0	0	1	100.0		1	100.0	0	0	
	6 days or more	3	0.9	2	66.7	1	33.3		1	33.3	2	66.7	
Days of using electronic cigarettes (last 30 days)								1					0.705
	Never	335	95.7	163	48.7	172	51.3		160	47.8	175	52.2	
	Less than 3 days	5	1.4	2	40.0	3	60.0		2	40.0	3	60.0	
	3 days or more	10	2.9	5	50.0	5	50.0		6	60.0	4	40.0	

**Table 9:** Bullying experiences, overall, by gender and school type.

				Gender					School Type				
		Overall (n = 350)		Female (n = 172)		Male (n = 180)			Public (n = 167)		Private (n = 185)		
		N	%	N	%	N	%	*p* -Value	N	%	N	%	*p*-Value
								0.114					0.800
Have you bullied someone at school?	No	314	89.7	157	50.0	157	50.0		150	47.8	164	52.2	
	Yes	36	10.3	13	36.1	23	63.9		18	50.0	18	50.0	
Have you bullied someone outside school?								0.333					0.832
	No	324	92.6	155	47.8	169	52.2		155	47.8	169	52.2	
	Yes	26	7.4	15	57.7	11	42.3		13	50.0	13	50.0	
Have you performed cyberbullying?								0.698					0.319
	No	337	96.3	163	48.4	174	51.6		160	47.5	177	52.5	
	Yes	13	3.7	7	53.8	6	46.2		8	61.5	5	38.5	
Who mostly bullied you?								0.902					0.053
	I was not bullied before	268	76.6	130	48.5	138	51.5		132	49.3	136	50.7	
	Students from my school	43	12.3	22	51.2	21	48.8		24	55.8	19	44.2	
	Someone else about my age	39	11.1	18	46.2	21	53.8		12	30.8	27	69.2	
Bullying method (face-to-face)								0.403					0.185
	I was not bullied face-to-face during the past 12 months	280	80.0	138	49.3	142	50.7		135	48.2	145	51.8	
	I was hit, kicked, pushed, shoved around, or locked indoors	2	0.6	1	50.0	1	50.0		1	50.0	1	50.0	
	I was made fun of, called a bad name, or teased	38	10.9	14	36.8	24	63.2		17	44.7	21	55.3	
	I was purposely ignored or left out of a group or activities	6	1.7	4	66.7	2	33.3		5	83.3	1	16.7	
	Someone spread rumors about me	5	1.4	4	80.0	1	20.0		4	80.0	1	20.0	
	Other	19	5.4	9	47.4	10	52.6		6	31.6	13	68.4	
Bullying methods (cyberbullying)								0.094					0.373
	I was not cyberbullied during the past 12 months	343	98.0	164	47.8	179	52.2		167	48.7	176	51.3	
	I was purposely ignored or left out of a group or activities online	1	0.3	1	100.0	0	0		0	0	1	100.0	
	Nasty or hurtful messages, pictures, or videos were sent to me	1	0.3	1	100.0	0	0		0	0	1	100.0	
	Harmful messages, photos or videos shared or posted online where others could see	0	0	0	0	0	0		0	0	0	0	
	Other ways	5	1.4	4	80.0	1	20.0		1	20.0	4	80.0	
Reason for bullying								0.688					0.515
	I was not bullied	281	80.5	139	49.5	142	50.5		137	48.8	144	51.2	
	Because of how my body or face looks	31	8.9	15	48.4	16	51.6		11	35.5	20	64.5	
	Because of how rich or poor my family is	2	0.6	1	50.0	1	50.0		1	50.0	1	50.0	
	Because of my religion	1	0.3	1	100.0	0	0		1	100.0	0	0.0	
	Some other reason	34	9.7	13	38.2	21	61.8		17	50.0	17	50.0	

## Data Availability

The original contributions presented in the study are included in the article, further inquiries can be directed to the corresponding author.
